# Morphology Reveals the Unexpected Cryptic Diversity in *Ceratophyllus gallinae* (Schrank, 1803) Infested *Cyanistes caeruleus* Linnaeus, 1758 Nest Boxes

**DOI:** 10.1007/s11686-020-00239-6

**Published:** 2020-06-08

**Authors:** Olga Pawełczyk, Tomasz Postawa, Marian Blaski, Krzysztof Solarz

**Affiliations:** 1 Department of Parasitology, Faculty of Pharmaceutical Sciences in Sosnowiec, Medical University of Silesia, Jedności 8, 41-200 Sosnowiec, Poland; 2 Department of Vertebrate Zoology, Institute of Systematics and Evolution of Animals of the Polish Academy of Sciences, Sławkowska 17, 31-016 Kraków, Poland; 3 Department of Zoology, Faculty of Biology and Environmental Protection, University of Silesia, Bankowa 9, 40-007 Katowice, Poland

**Keywords:** Body size variability, *Ceratophyllus gallinae*, Fleas, Morphological diversity, Sexual size dimorphism

## Abstract

**Purpose:**

The main aim of our study was to examine morphological differentiation between and within sex of hen fleas—*Ceratophyllus gallinae* (Schrank, 1803) population collected from Eurasian blue tit (*Cyanistes caeruleus* Linnaeus, 1758), inhabiting nest boxes and to determine the morphological parameters differentiating this population.

**Methods:**

A total of 296 fleas were collected (148 females and 148 males), determined to species and sex, then the following characters were measured in each of the examined fleas: body length, body width, length of head, width of head, length of comb, height of comb, length of tarsus, length of thorax and length of abdomen.

**Results:**

The comparison of body size showed the presence of two groups among female and male life forms of the hen flea, which mostly differed in length of abdomen, whereas the length of head and tarsus III were less variable.

**Conclusion:**

Till now, the only certain information is the presence of two adult life forms of *C. gallinae*. The genesis of their creation is still unknown and we are not able to identify the mechanism responsible for the morphological differentiation of fleas collected from the same host. In order to find answer to this question, future research in the field of molecular taxonomy is required.

## Introduction

Females and males of nearly all arthropods differ in their body size. This phenomenon is also widespread among insects and is called a sexual size dimorphism (SSD). SSD likely appears when growth patterns of females and males of the same species differ in their sensitivity to environmental conditions [1]. In addition, the larger size of body and particular body parts is more desirable among insects. The main reasons of larger size selection may be the increase of mating success in males and fecundity of females, what allows to produce a bigger offspring [1–8]. Moreover, seasonal changes of environment and presence of many differences between life forms during arthropods’ life cycle can cause the phenotype variation among adult individuals [8, 9]. They may differ in shape, behavior and their response to environmental factors. Heteromorphism—different forms at various periods of the male life cycle, is a phenomenon known among Acari, including groups of mites such as Astigmata and Prostigmata [10–14]. It is often related to sexual behavior or male reproductive strategy and can lead to increased reproductive success among heteromorphic males [11, 13]. In turn, gynopolymorphism (sex carrying the polymorphism in female) was reported among representatives of order Diptera (*Drosophila melanogaster*), Odonata (*Coenagrion*, *Enallagma*, *Ischnura*) and many species of Lepidoptera (e.g. from *Colias* genus) [15–17].

Variability of ectoparasite population is caused by degree of host specificity, the mode of parasite transmission and the ability for adaptive phenotypic plasticity [18, 19]. Fleas are obligatory external parasites of birds and mammals, thus their more pronounced morphological variability can depend on their type of the host and the host living environment. Individual morphological characteristics of fleas can be a result of host migration, its diet or condition [9, 20]. External parasites are also subjected to a strong selection, which can lead to convergent evolved phenotypes among one or more species. Furthermore, a strong selective pressure on loci, which controls morphological differences in organisms, can be caused by genetic isolation of the population of each host and the adaptation to a particular host [19, 21].

Fleas demonstrate strong female-biased SSD, what is a common trend among other groups of arthropods. Sexual size dimorphism decreases in larger species [22]. Furthermore, fleas do not demonstrate strong stable selection or strong constraints on female size. For example, egg production and egg size in fleas are reported to be independent of body size [23, 24], but were explained well by the patterns of relationships with their hosts. A high morphological variability between males and females was reported in *Ceratophyllus hirundinis* (Curtis 1826) collected from nests of *Delichon urbica* (Linnaeus 1758) (Upper Silesia, Poland) [25]. Similar pattern was observed in a morphological variability of *Ceratophyllus gallinae* (Schrank 1803) collected from *Cyanistes caeruleus* Linnaeus 1758 (before *Parus caeruleus*) nests. These results demonstrate that both males and females manifested a high morphological variability [9].

Main aims of our study are to examine the morphological differentiation between and within sex of population collected from Eurasian Blue Tits *C. caeruleus* inhabiting nest boxes and to determine morphological parameters differentiating this population.

## Materials and Methods

### Ectoparasite Species

The hen flea *C. gallinae* is a common species in Europe, North America and New Zealand, which has an extremely wide range of parasitizing hosts including 72 wild birds’ species and about 15 species of mammals. This flea regularly invades poultry and could cause the fatal iron deficiency, anemia and allergic dermatitis of fowls [26–28]. To the main hosts of *C. gallinae* belong blue (*C. caeruleus*) and great (*Parus major*) tits [28, 29]. Most of *C. gallinae* adults overwintering in the cocoons and occur in relatively large numbers in nests of their hosts during winter. In contrast to the birds breeding season, when fleas disperse among birds generations [30, 31]. *C. gallinae* fleas often occur with another flea species—*Dasypsyllus g. gallinulae* (Dale, 1878) or with blow flies of the genus *Protocalliphora* (Diptera: Calliphoridae) in the same nest, which can affect bird reproductive performance [31, 32].

### Host Species

In this study, fleas were collected from the blue tits *C. caeruleus* nest boxes. Blue tits are common European passerine birds, which build their nests in natural tree-holes and often inhabit nest boxes. Each spring, females lay around 10 eggs, which are incubated and hatch 2 weeks later. *C. gallinae* infestation in nests and nest boxes significantly affects the behavior of bird females [28, 33, 34].

### Data Collection and Material Preparation

The hen fleas *C. gallinae* were collected in the period between 1998–2003 from blue tits (*C. caeruleus*) nest boxes, which occurred in Panewniki Forestry Commission, land in the vicinity of Ruda Śląska (Upper Silesia, in the Southern Poland). Nest boxes were placed in deciduous forest of Panewniki Forestry Commission, and systematically examined throughout the year except for May, when tits lay eggs and nestlings hatch. Nest boxes and fleas were not collected at that time, in order to protect birds. All fleas were isolated from the nests and litter collected from nest boxes by using Tullgren apparatus. All specimens were preserved in 70% ethyl alcohol and preparated. Fleas were placed on microscopic permanent slides in Berlese solution. *C. gallinae* were determined according to Skuratowicz key [26]. All specimens were deposited in Department of Zoology at the University of Silesia (Katowice, Poland). A total of 296 fleas were collected, including 148 females and 148 males. To determine fleas to species, sex and to measure morphological features optical microscopy were used (Olympus CH40). The following characters were measured in each of the examined fleas: body length, body width, length of head, width of head, length of comb, height of comb, length of tarsus, length of thorax and length of abdomen. The examined material has been clustered into males and females, and after preliminary morphometric analysis into males and females with a typical and smaller body size for examined flea species. SSD-related traits are length and height of comb. Moreover, the body length, body width, length of thorax, length of abdomen, length of head and width of head could be determined by SSD. These six features could be also determined by the nutritional status of individual. The length of tarsus depends on physiological differences, such as jumping performance [35].

### Data Analysis

Data were analyzed in several steps. Initially, sex determination and morphological measurements specimens were performed. Due to presence of noticeable differences in their size: normal type and smaller type, specimens were preliminary divided into two subgroups. The “small” and “typical” forms were previously marked by Blaski et al. [9]. The “typical” forms had bigger size of thorax and abdomen than “smaller” forms, while size of head and tarsus in both were similar. To test whether this classification is valid, morphometric data (continuous variables) were subjected to hierarchical cluster analysis with the Ward’s minimum variance method (clustering tree: R package pvclust) [36]. Obtained separate clusters with addition of sex effect were analyzed using a two-way Multivariate Analysis of Variance (MANOVA). Post hoc comparisons were conducted to test for differences between the two forms and sex in each morphological measurement. We compared variation within and between obtained groups using MANOVA with fixed factor: sex and form of flea. Analyses were performed using R (R software v. 3.4.3) [37]. In all tests, values of *p* < 0.05 were considered significant.

## Results

**Fig. 1:**
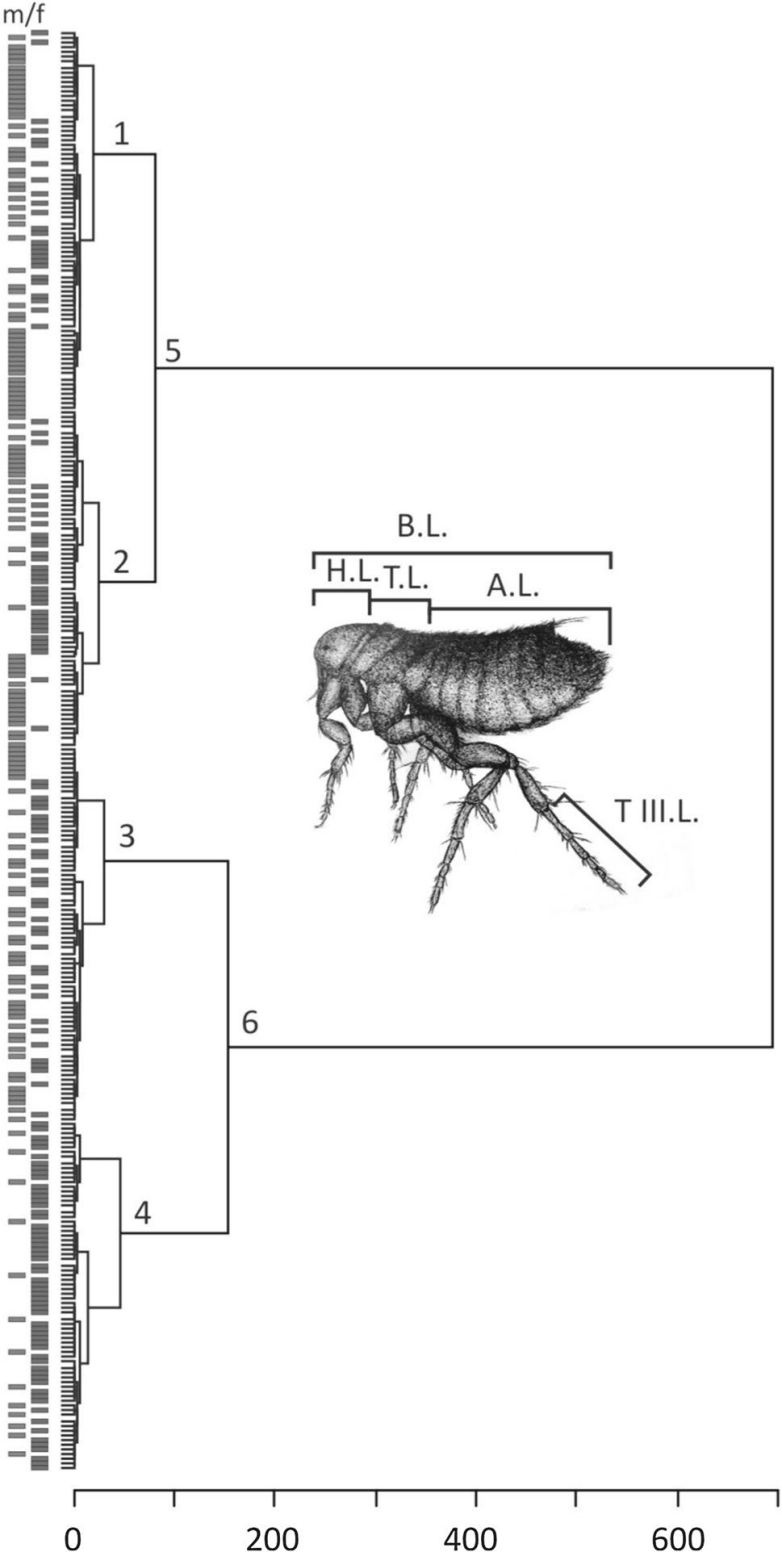
Hierarchical cluster analysis (Ward’s methods) of flea specimens (*n* = 296) using morphometrics traits. *B.L.* body length; *H.L.* head length; *T.L.* thorax length; *A.L.* abdomen length; *T III. L.* tarsus III length, *m* males, *f* females

Hierarchical clustering analysis based on nine morphometrical body size parameters of *C. gallinae* (*n* = 296) individuals tightly corresponded with the pre-selection determination and consists from two subgroups. Cluster 5 corresponded with smaller form (males = 90, females = 57), while cluster 6–with normal size form (males = 58, females = 91) (Fig. 1). The two groups indicated by cluster analysis were completely identical to the preliminary classification in “small” and “typical”. Remaining clusters: 1, 2, 3 and 4 were not clearly separated based on sex, but rather exhibited intermorphotype variability, therefore were excluded from further analyses.

This differentiation was supported by a multivariate analysis of variance (MANOVA). The two main clusters (5 and 6) differed significantly in analyzed morphological traits of the body size (MANOVA, Wilk’s *λ* = 0.163, *d.f.* = 9,284, *F* = 161.0, *p* < 0.0001), the differences were also found between sexes (Wilk’s *λ* = 0.470, *d.f.* = 9,284, *F* = 35.7, *p* < 0.0001). The interaction and sex clusters were significantly different (Wilk’s *λ* = 0.902, *d.f.* = 9,284, *F* = 3.40, *p* = 0.0005). Variances were explained by length of abdomen, body length and body width, less by length of thorax, width of head, height of comb, and in minor degree by length of tarsus, length of comb and length of head (Table 1).

**Table 1:** Results of the subsequent analysis of variance (two-way ANOVA-s) showing the morphometrical traits distinguishing the “typical” and the “small” form of *Ceratophyllus gallinae*

Character	Factor	*d.f*	*F*	*p*
Length of body	Cluster	1	927.1	< 0.00001***
	Sex	1	29.2	< 0.00001***
	Cluster × sex	1	10.0	0.002**
	Residuals	292		
	*R*^2^ adjusted = 76.6%			
Width of body	Cluster	1	442.5	< 0.00001***
	Sex	1	106.3	< 0.00001***
	Cluster × sex	1	2.93	0.09
	Residuals	292		
	*R*^2^ adjusted = 65.0%			
Length of head	Cluster	1	7.35	0.007**
	Sex	1	1.36	0.245
	Cluster × sex	1	7.68	0.006**
	Residuals	292		
	*R*^2^ adjusted = 43.4%			
Width of head	Cluster	1	79.4	< 0.00001***
	Sex	1	171.1	< 0.00001***
	Cluster × sex	1	0.35	0.56
	Residuals	292		
	*R*^2^ adjusted = 45.6%			
Length of pronotal comb (ctenidium)	Cluster	1	23.04	< 0.00001***
	Sex	1	50.3	< 0.00001***
	Cluster × sex	1	0.67	0.41
	Residuals	292		
	*R*^2^ adjusted = 19.4%			
Height of pronotal comb (ctenidium)	Cluster	1	58.3	< 0.00001***
	Sex	1	97.0	< 0.00001***
	Cluster × sex	1	0.52	0.47
	Residuals	292		
	*R*^2^ adjusted = 34.1%			
Length of tarsus III	Cluster	1	56.3	< 0.00001***
	Sex	1	69.8	< 0.00001***
	Cluster × sex	1	0.294	0.56
	Residuals	292		
	*R*^2^ adjusted = 29.5%			
Length of thorax	Cluster	1	199.3	< 0.00001***
	Sex	1	66.2	< 0.00001***
	Cluster × sex	1	0.342	0.56
	Residuals	292		
	*R*^2^ adjusted = 47.0%			
Length of abdomen	Cluster	1	1091.8	< 0.00001***
	Sex	1	14.6	0.0002***
	Cluster × sex	1	16.7	0.00006***
	Residuals	292		
	*R*^2^ adjusted = 79.2%			

*d.f.* degrees of freedom

*p* statistical significance (*p < 0.05, **p < 0.01, ***p < 0.001)

**Fig. 2:**
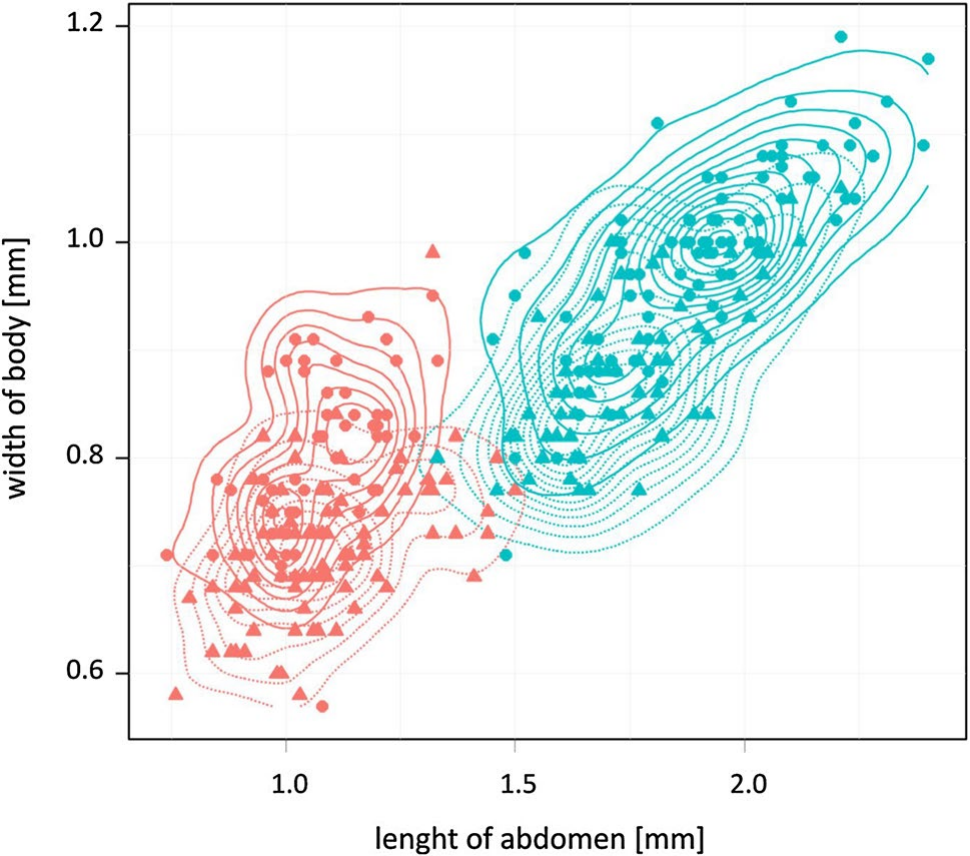
Scatter plot of abdomen length against body width of *Ceratophyllus gallinae*. *Triangle*—males, *filled circle*—females; Density lines: *turquoise*—“typical” form, *orange*—“small” form, *solid line*—females, *dashed lines*—males

Size differences between the forms are determined by all nine analyzed parameters, the most divergent measurement: length of abdomen and body length, following length of thorax, body width, with minor differences in remaining parameters: width of head, height and length of comb, length of tarsus and length of head (Table 1). Sex differences (within forms) were smaller, and accounted from 1 to almost 18%. Among eight analyzed parameters the most differentiate were width of head, length of thorax, height and length of comb, body width and length of tarsus III. In turn, minor differences were found in body length, length of abdomen and length of head. Interaction cluster and sex, which indicated differences between clusters in size alter with sex, were noted in three analyzed parameters, such as body length, body width and length of abdomen (Table 2). Therefore, the differences between clusters (forms) appeared to be homogeneous. The mutual dependencies of abdomen length and body width of examined *C. gallinae* individuals show a positive correlation (Fig. 2).

**Table 2:** Mean (± SD) and confidence interval (± 95%) of body size two population of adult hen flea *Ceratophyllus gallinae*: “typical and, small” form. Two-way ANOVA was used to evaluate statistically significant differences between the two clusters and their sex

Males	Typical form (*n* = 58)			Small form (*n* = 90)					
Character	Mean	SD	± 95%	Mean	SD	± 95%	*F*	*p*	
Body length	2.54	0.235	2.48–2.60	1.77	0.174	1.731–1.804	375.1	< 0.00001	***
Body width	0.88	0.075	0.86–0.90	0.72	0.064	0.704–0.731	154.2	< 0.00001	***
Head length	0.35	0.029	0.35–0.36	0.34	0.031	0.331–0.344	16.4	0.0001	***
Head width	0.25	0.036	0.24–0.26	0.23	0.027	0.225–0.237	15.5	0.0001	***
Pronotal comb length	0.083	0.013	0.08–0.09	0.078	0.013	0.075–0.080	2.64	0.106	ns
Pronotal comb height	0.23	0.027	0.22–0.23	0.21	0.027	0.202–0.213	10.5	0.0015	**
Tarsus III length	0.80	0.062	0.79–0.82	0.75	0.058	0.741–0.766	15.6	0.0001	***
Thorax length	0.43	0.061	0.41–0.44	0.34	0.046	0.327–0.346	77.1	< 0.00001	***
Abdomen length	1.74	0.178	1.69–1.79	1.08	0.163	1.048–1.117	376.4	< 0.00001	***

Females	Typical form (*n* = 92)			Small form (*n* = 56)					
Character	Mean	SD	± 95%	Mean	SD	± 95%	*F*	*p*	
Body length	2.77	0.298	2.710–2.834	1.83	0.166	1.787–1.876	437.1	< 0.00001	***
Body width	0.98	0.087	0.967–1.003	0.80	0.076	0.779–0.820	177.5	< 0.00001	***
Head length	0.34	0.029	0.336–0.348	0.34	0.032	0.334–0.351	0.01	0.931	ns
Head width	0.31	0.035	0.299–0.313	0.28	0.036	0.270–0.289	17.8	0.00004	***
Pronotal comb length	0.094	0.013	0.091–0.097	0.088	0.014	0.084–0.092	7.56	0.007	**
Pronotal comb height	0.26	0.029	0.253–0.265	0.24	0.027	0.232–0.246	17.4	0.00005	***
Tarsus III length	0.87	0.073	0.856–0.887	0.82	0.082	0.800–0.844	14.7	0.0002	***
Thorax length	0.49	0.069	0.471–0.500	0.39	0.057	0.377–0.408	67.5	< 0.00001	***
Abdomen length	1.91	0.214	1.861–1.950	1.07	0.122	1.041–1.106	621.7	< 0.00001	***

*SD* standard deviation,  ± 95% confidence interval

*p* statistical significance (*p < 0.05, **p < 0.01, ***p < 0.001), *ns* not significant

## Discussion

This study highlights the presence of differences both in SSD and various morphotypes (“small” and “typical”) among males and females of examined *C. gallinae* population. Theory would predict that in organisms often exposed to variation in food resource during development, adaptive plasticity would evolve to minimize such effects [38, 39]. This prediction is also supported by the observation of highly orchestrated development of different body parts and organs in holometabolous insects developing under different food qualities [40, 41]. However, if the variation in food resource is highly unpredictable, such adaptive plasticity is less likely to evolve [42]. Thus, adaptive intraspecific allometry of life span and reproduction is expected, when variation in body size is genetic or environmentally induced, as long as there are reliable cues to be used enabling the organism to predict the variation in food resource, but to less extent when variation in growth conditions is unpredictable [43].

The comparison of body size showed that the examined forms in the population mostly differ in length of abdomen, whereas length of head and tarsus III were less variable. Similar results found Blaski et al. [9] analyzing seasonal morphological variability of *C. gallinae* parasitized blue tits. That study also highlights the presence of two groups among male and female life forms. The occurrence of two morphologically different adult forms in other species from Siphonaptera is still not found, therefore the hen flea is the only known species, which showed a peculiar type of morphological diversity [9, 25, 34]. In our study, two different flea morphotypes occurred together in the same nest boxes, regardless of the season when they were collected.

Males and females of fleas, which parasitize on the same hosts and share the same alimentary niche, undergo natural selection [22]. If the primary host spends most of the time in the nest, like in case of *C. caeruleus* the variability of environmental conditions is more observable. Moreover, the differences in morphology among individuals of one flea species can depend on microhabitat, where larval development occurs and adult forms exist [28]. In 1999, Tripet and Richner [34] were modulating natural conditions in blue tits nests and analyzed the dynamics of hen fleas in order to check their interactions. During one birds’ nestling cycle, they observed presence of two different adult forms and named them as subpopulations. In regards to the fact, that the examined material in this study came from identical nest boxes and the conditions were not modulated, we can assume that physiochemical conditions were very similar. Thus we could imply our results to a foregoing study. There are suggestions about the existence of local adaptation between hen fleas and great tits [44]. It based on observation of reproductive success *C. gallinae*, which at the end of tits breeding was higher for foreign than local fleas. It could be parasite local maladaptation, but on the other hand, reproductive success of great tits was lower for nests infested by foreign fleas in comparison to the control (nests infested by local fleas in an intermediate position) [44].

In general, bimodal variance of body size is rarely encountered, and even unparallel in case of sexual dimorphism. This phenomenon among one species is often a basis to separation of a taxon, but sometimes like in the study from 2013 by Balvin et al. [19], two of those forms were found genetically identical (Cimicidae). The mentioned study showed that populations of haematophagous ectoparasites—*Cimex pipistrelli* Jenyns, 1839 bat bugs were morphologically differentiated between host species, but that variability was not supported by nuclear and mitochondrial DNA [19].

In conclusion, the only certain information is the presence of two adult life forms of *C. gallinae*. We know that development of two adult forms of this species is probably not dependent on the type of host or season [9]. The genesis of their creation is still unknown and we are not able to identify the mechanism responsible for the morphological differentiation of fleas collected from the same host. In order to find the answer to this question, future research in the field of molecular taxonomy is required. Presences of two morphologically different adult forms in other species from the order Siphonaptera until now were not found, therefore, *C. gallinae* is actually the only known species which showed a peculiar type of morphological diversity.
